# Differences between Scheimpflug and optical coherence tomography in determining safety distances in eyes with an iris-fixating phakic intraocular lens

**DOI:** 10.1007/s00417-020-04874-7

**Published:** 2020-08-06

**Authors:** Zoraida S. Gaurisankar, Gwyneth A. van Rijn, Gregorius P. M. Luyten, Jan-Willem M. Beenakker

**Affiliations:** 1 Department of Ophthalmology, Leiden University Medical Center, Leiden, the Netherlands; 2 Leiden, the Netherlands; 3 Department of Radiology, C.J. Gorter Center for High-Field MRI, Leiden University Medical Center, Leiden, the Netherlands

**Keywords:** Phakic intraocular lens, Lens position, PENTACAM, Scheimpflug, Visante OCT, Anterior segment OCT

## Abstract

**Purpose:**

To investigate the agreement and reliability of anterior segment optical coherence tomography (AS-OCT) and Scheimpflug imaging in measuring the distance from the anterior edge of an iris-fixated phakic intraocular lens (IF-pIOL) to the corneal endothelium.

**Methods:**

Anterior segment configuration was assessed in a total of 62 eyes of which 25 hyperopic and 37 myopic eyes, all corrected with an IF-pIOL. Measurements were performed by two independent observers using AS-OCT (Visante, Model 1000, Carl Zeiss Meditec Inc.) and Scheimpflug imaging (Pentacam HR, Oculus Optikgerate). The distance from the anterior edge of the pIOL to the endothelium was measured in five different positions using both modalities with their corresponding pIOL software. The measurements as well as the inter- and intra-observer reliability of the two imaging modalities were then compared.

**Results:**

Distance measurements for all positions performed by AS-OCT were found to be significantly larger than those performed by Scheimpflug imaging, with mean differences ranging from 0.11 to 0.22 mm. Both instruments exhibited good inter- and intra-observer reliability.

**Conclusion:**

Anterior pIOL edge to endothelium distance measurements by AS-OCT and Scheimpflug imaging have good intra- and inter-observer reliability. However, as AS-OCT provides larger measurements, these two modalities cannot be used interchangeably. Correction of this difference might be essential for proper decision-making during pre-operative screening for pIOL implantation and post-operative safety monitoring.

**Electronic supplementary material:**

The online version of this article (10.1007/s00417-020-04874-7) contains supplementary material, which is available to authorized users.

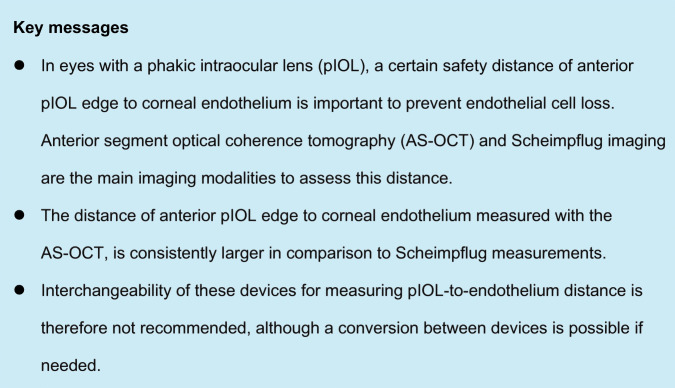



## Introduction

Phakic intraocular lens (pIOL) implantation has proven to be safe and effective for the correction of a broad range of ametropia [1, 2]. The Artisan lens (Ophtec BV, Groningen, the Netherlands) is an iris-fixated (IF) pIOL that has been used successfully to correct moderate to high myopia, hyperopia, and astigmatism since 1991. The outcomes after Artisan implantation have found to be predictive and stable over time [1, 3, 4].

To establish the long-term safety of IF-pIOL and to prevent complications, an extensive pre-operative evaluation in combination with long-term post-operative follow-up is required. One of the most feared and important potential complications of any type of anterior segment surgery is accelerated endothelial cell (EC) loss, especially in the case of IF-pIOL. As this risk has been shown to be negatively correlated to the anterior chamber depth, the position of an IF-pIOL in the anterior chamber is one of the main safety parameters in both pre-operative screening and follow-up [1, 4–9].

Monitoring of the anatomical relationship with an IF-pIOL in the eye can be performed at the slit lamp. However, the accuracy between the distance of the pIOL to the corneal endothelium is subject to subjective interpretation and is thus limited. To objectively measure the distance between the central and peripheral pIOL edge to the corneal endothelium, several clinical techniques may be used, including ultrasound biomicroscopy (UBM), Scheimpflug imaging, and anterior segment optical coherence tomography (AS-OCT). UBM delivers images of excellent quality but has several limitations, such as the fact that it is technically challenging, with a risk of distorting the true anterior chamber dimension, time-consuming to perform, and possibly uncomfortable for the patients [10]. The non-contact AS-OCT [11–13] and Scheimpflug imaging techniques [14–16] both provide high-resolution images of the anterior chamber on which the pIOL position can be determined with the provided software.

To minimize the risk of increased cell loss, Baïkoff introduced in 2006 the “minimum (or ‘critical’) safety distance”: a minimum distance between the central edge of the optical zone of the pIOL and the endothelium [11]. Based on the clinical results of Pérez-Santonja et al. [17] and de Sousa et al. [18], he proposed a minimum distance of 1.5 mm to prevent accelerated EC loss. Later studies confirmed the importance of the central distance between the anterior surface of the pIOL and the endothelium [13, 15, 16, 19], showing a yearly increase in EC loss with smaller distances. Doors et al. described an average EC loss of 0.15%, 0.98%, and 1.80% per year for a minimum central distance between the anterior surface of the pIOL and the endothelium of 1.59 mm, 1.37 mm, and 1.15 mm, respectively [13]. In addition to the central distances and a smaller ACD, Jonker et al. [19] found smaller distances between the peripheral pIOL edge and endothelium to also be a significant risk factor for accelerated EC loss.

The aim of this study is to compare the AS-OCT and Scheimpflug imaging in measuring pIOL-to-endothelium distances and to assess the inter- and intra-observer variability of these measurements.

## Methods

In this cross-sectional study, we examined 62 phakic eyes that had undergone pIOL implantation, of which 25 eyes (13 patients) were corrected for hyperopia and 37 eyes (20 patients) for myopia. All the eyes were implanted with an Artisan IF-pIOL by the same experienced eye surgeon at the Leiden University Medical Center (LUMC), Leiden, or Erasmus Medical Center, Rotterdam; Artisan lens model 203 was implanted for hyperopia and model 206 for myopia, with the available refractive powers ranging from + 1.0 to + 12.0 diopters and − 1.0 to − 23.5 diopters respectively, in 0.5 diopter steps. The study was approved by the Medical Ethical Committee of the LUMC and was conducted in accordance with the Declaration of Helsinki. Informed consent was obtained from all patients before they were examined. Anterior segment scans were made with two different imaging modalities: the AS-OCT and Scheimpflug imaging. All images were made under the same dim light conditions in an unaccommodated state.

The Visante OCT (Visante, Model 1000, software version 3.0.1.8, Carl Zeiss Meditec Inc.) is a time-domain system that uses infrared light (1310 nm) to image the anterior segment. For this study, all measurements were performed in high-resolution mode, which provides a detailed image with a field of view of 10 mm width by 3 mm. In this mode, the Visante performs 512 scans to assess the anterior segment area in 0.25 s. Axial and transverse resolutions are 18 and 60 μm, respectively.

The Pentacam HR system (Pentacam HR, software version 1.12r24, Oculus Optikgerate) uses the Scheimpflug imaging technique for anterior segment evaluation. A 360°, rotating, non-contact camera uses a monochromatic slit light source to reconstruct a three-dimensional map of the anterior segment of the eye. Such a scan is performed in 2 s and yields images with a clear visualization of the pIOL. For assessing the pIOL position, a 3-D pIOL-simulation software module is provided.

**Fig. 1:**
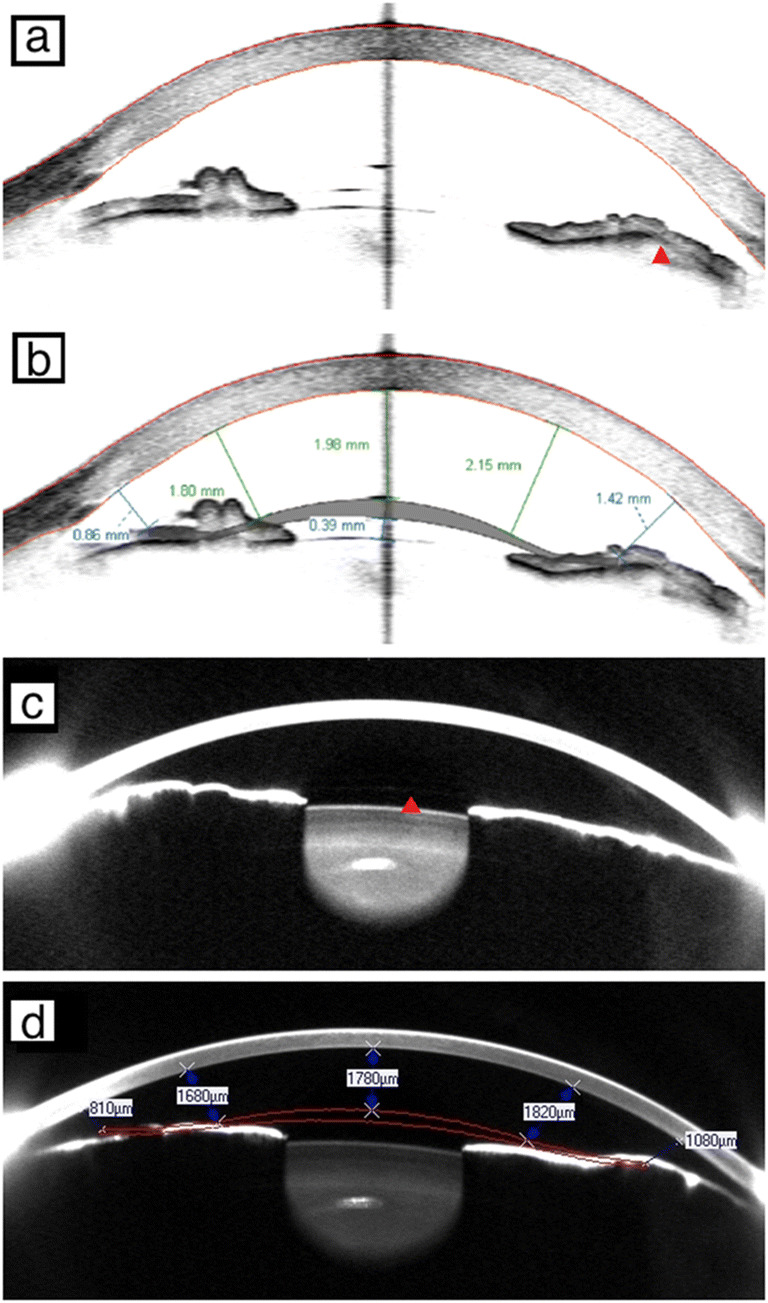
Anterior segment scan image acquired with the Visante anterior segment optical coherence tomography (AS-OCT) before (**a**; *red arrow*: phakic intraocular lens (pIOL) enclavation site) and after placement of the pIOL template using the pIOL analysis software (**b**). Similar images acquired with Scheimpflug imaging before (**c**; *red arrow*: edge of pIOL) and after placement of pIOL template (**d**; contrast of scan was adjusted). All four scans represent the left eye of the same subject on a 180°–0° axis. (Please note the differences in clearance distances given by the Pentacam compared with the Visante)

The acquired images were subsequently analyzed using the vendors’ software. With AS-OCT, the distance from the pIOL to the corneal endothelium is measured by manually placing a pIOL template on the anterior segment image by computer mouse selection and dragging and drawing a measurement vector using the vendor’s software (Fig. 1a, b). In the case of Scheimpflug imaging, the software automatically calculates the minimum distance between the pIOL and the corneal endothelium after the 3-D pIOL template is manually added to the image (Fig. 1c, d). When present, the iris image is used for better precision of the pIOL template position. On both types of anterior segment scans, the pIOL-to-endothelium distance was measured in five standard positions along the 180° horizontal axis (at “3 o’clock” and “9 o’clock” positions) (Fig. 1b, d):

- Central
- At 2.5 mm nasal from the center
- At 2.5 mm temporal from the center
- At 4 mm nasal from the center
- At 4 mm temporal from the center

To determine the inter- and intra-observer variability, these analyses were performed separately by two independent, trained observers (ZSG, GAR). Both observers repeated the measurements at another time point, at least 3 months from the first measurements and without knowledge of the earlier results. To test the agreement between the two imaging modalities, the average of all four measurements was used for analysis.

### Statistical analysis

All statistical analyses were performed using SPSS statistics software version 25 (SPSS Inc., IBM, Somers, NY).

To assess the agreement between tomographers, a paired sample *t* test was applied and the Bland–Altman analysis was performed, and 95% limits of agreement (LoA) were estimated by the mean difference ± 1.96 × standard deviation (SD) of the difference [20]. To exclude potential cofounding factors (right or left eye, hyperopic or myopic eye, time interval between pIOL implantation and examination date), a linear mixed model was used where these factors were taken into account to test their significance. A *p* value of < 0.05 was considered to be statistically significant.

Inter- and intra-observer reliability was assessed by calculating the intra-class correlation coefficients (ICC) using a multilevel (hierarchical) linear mixed model to adjust for the possible correlation between measurements within the same eye and between the two eyes within the same patient. In this model, intra-observer reliability was evaluated by correlating each observer’s first measurement by AS-OCT and Scheimpflug imaging with the same observer’s second measurement. Inter-observer reliability was assessed by correlating measurements of one observer with the corresponding measurements of the other observer. The ICC was interpreted according to Cohen’s kappa classification [21].

## Results

### Patient characteristics

Sixty-two phakic eyes of 34 subjects including 11 males and 23 females between the age of 24.9 and 76.6 years, with a mean (SD) of 49.6 (11.2) years, were examined. The power of the Artisan lenses implanted ranged from + 12.00 to − 23.50 diopters. The mean time interval between pIOL implantation and the first anterior segment analysis was 9.7 (4.7) years. For more details, see Table 1.

**Table 1:** Patient characteristics

Variable	Total	Hyperopic eyes	Myopic eyes
Eyes (count)	62 32:68	25 (12 right eyes) 64:36	37 (17 right eyes) 11:89
Sex (male:female) [%]			
Age at examination ± SD (min–max) [years] pIOL power ± SD (min–max) [D] Time interval between pIOL implantation and anterior segment examination ± SD (min–max) [years]	49.6 ± 11.2 (24.9–76.6) 9.7 ± 4.7 (0.0–18.0)	52.6 ± 9.3 (24.9–67.4) 7.7 ± 2.6 (2.0–12.0) 9.8 ± 3.6 (0.0-14.0)	47.6 ± 12.0 (25.9–76.6) − 13.6 ± 4.6 (− 23.5 to − 13.6) 9.5 ± 5.5 (0.0–18.0)

*SD* standard deviation; *pIOL* phakic intraocular lens; *D* diopters

### Inter- and intra-observer reliability

The overall *inter*-observer ICC was 0.99 with a 95% confidence interval (CI) of 0.99−0.99 for both AS-OCT and Scheimpflug imaging. The overall *intra*-observer ICC was 0.99 with a 95% CI of 0.99−0.99 for AS-OCT and 0.98 with a 95% CI of 0.98−0.98 for Scheimpflug imaging. The ICCs per position measurement of each instrument are shown in Table 2. All correlations were “very good” for both AS-OCT and Scheimpflug imaging according to Cohen’s kappa classification [21], showing that a single measurement is reliable irrespective of observer or measurement occasion.

**Table 2:** Intra-class correlation coefficients of anterior segment optical coherence tomography and Scheimpflug imaging show good reproducibility of analysis for both modalities

	AS-OCT		Scheimpflug imaging	
	ICC		ICC	
	Inter-observer (95% CI)	Intra-observer (95% CI)	Inter-observer (95% CI)	Intra-observer (95% CI)
4.0 mm nasal endothelium to pIOL	0.944 (0.908–0.966)	0.917 (0.882–0.942)	0.890 (0.813–0.935)	0.818 (0.740–0.873)
2.5 mm nasal endothelium to pIOL	0.969 (0.949–0.982)	0.961 (0.944–0.972)	0.958 (0.928–0.976)	0.913 (0.875–0.939)
central endothelium to pIOL	0.996 (0.994–0.998)	0.909 (0.835–0.949)	0.955 (0.910–0.976)	0.991 (0.987–0.994)
2.5 mm temporal endothelium to pIOL	0.946 (0.911–0.968)	0.930 (0.901–0.951)	0.965 (0.940–0.979)	0.944 (0.920–0.961)
4.0 mm temporal endothelium to pIOL	0.955 (0.910–0.976)	0.948 (0.926–0.964)	0.955 (0.920–0.974)	0.919 (0.884–0.944)

*AS-OCT* anterior segment optical coherence tomography; *ICC* intra-class correlation coefficient; *95% CI* 95% confidence interval; *pIOL* phakic intraocular lens

### Agreement between instruments

**Fig. 2:**
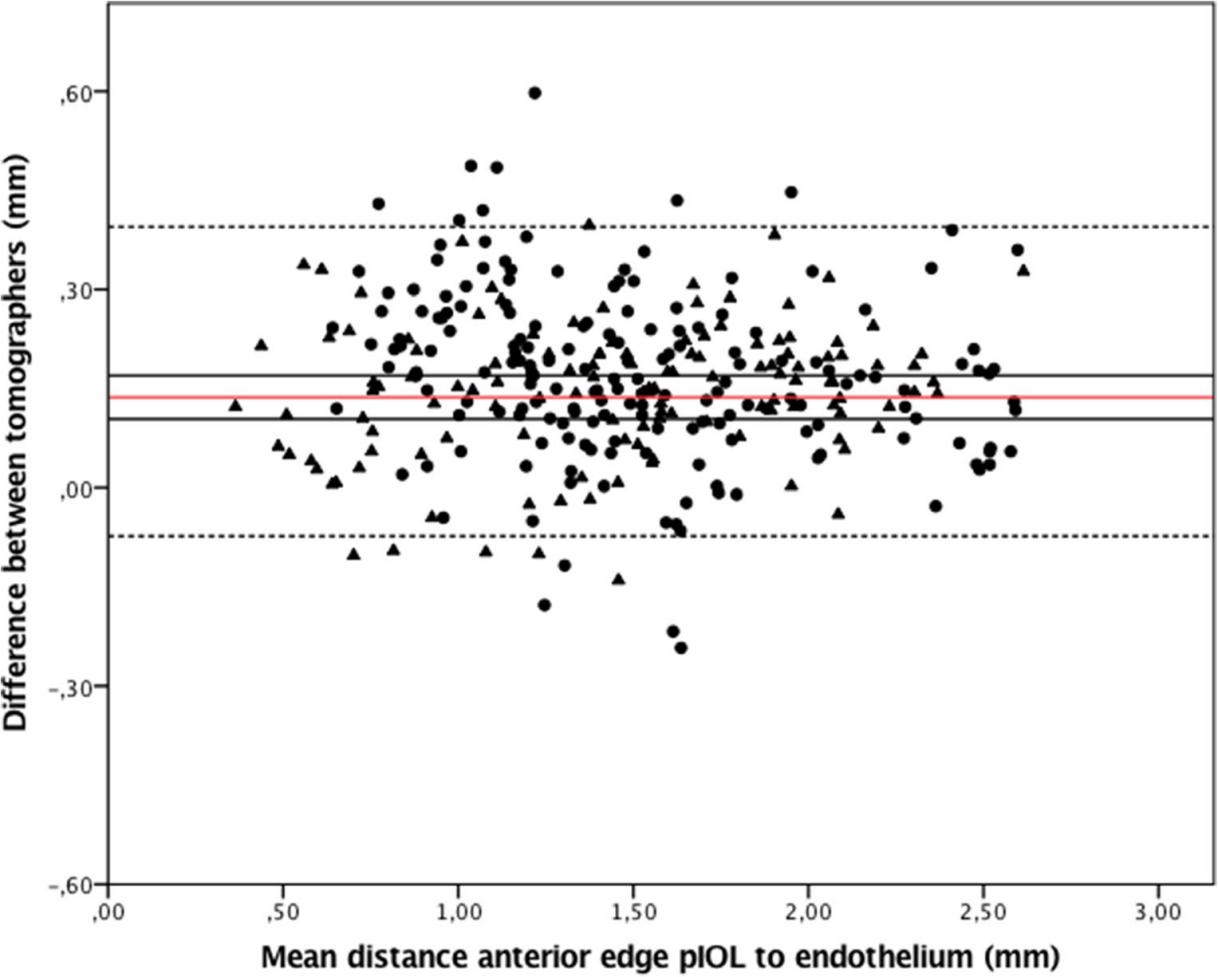
Bland-Altman plot showing the difference in distance measurements between the anterior segment optical coherence tomography and Scheimpflug imaging modalities for all positions from the anterior phakic intraocular lens (pIOL) to the endothelium. The red line represents the mean, the black line the upper and lower 95% confidence interval, the dashed lines the upper and lower 95% limits of agreement (LoA). *Triangles*: hyperopic eyes; *dots*: myopic eyes

The distance from the anterior edge of the pIOL to the endothelium when measured by AS-OCT was consistently larger than when measured by Scheimpflug imaging, for all five separate positions, as listed in Table 3. The mean difference for all of the various positions was 0.161 (0.120) mm with a 95% LoA of − 0.074 and 0.396 (paired *t*_309_ = 23.74; *p* < 0.001), see Fig. 2 for the Bland Altman plot. The peripheral measurements showed similar results. Supplementary Fig. 1 shows the Bland-Altman plots for the differences in distance measurements at the 5 positions with the 95% LoA and 95% CIs. The mean difference between AS-OCT and Scheimpflug imaging for the central distance measurements was 0.150 mm (95% LoA, − 0.014 and 0.314), for 2.5 mm nasal 0.189 mm (95% LoA, − 0.020 and 0.398), for 2.5 mm temporal 0.114 mm (95% LoA, − 0.102 and 0.330), for 4.0 mm nasal 0.218 mm (95% LoA, − 0.045 and 0.481), and for 4.0 mm temporal 0.137 mm (95% LoA, − 0.115 and 0.389). In a mixed model, distance measurements were not found to be significantly affected by age, sex, right or left eye, hyperopic or myopic eye, or the time interval between pIOL implantation and the examination date, so these factors were not included in further analyses.

**Table 3:** Means and differences in distance measurements made by anterior segment optical coherence tomography and Scheimpflug imaging

	AS-OCT	Scheimpflug	AS-OCT versus Scheimpflug			
Measurement to endothelium [mm] from	Mean ± SD	Mean ± SD	Difference (mean ± SD)	Range	95% CI	*p* value
4.0 mm nasal of anterior edge of pIOL	1.018 ± 0.249	0.799 ± 0.231	0.218 ± 0.135	0.30–1.52	0.184–0.253	< 0.001
2.5 mm nasal of anterior edge of pIOL center of anterior edge of pIOL 2.5 mm temporal of anterior edge of pIOL 4.0 mm temporal of anterior edge of pIOL All five positions of anterior edge of pIOL	1.652 ± 0.282 2.184 ± 0.361 1.760 ± 0.271 1.180 ± 0.280 1.509 ± 0.509	1.462 ± 0.251 2.034 ± 0.362 1.647 ± 0.261 1.043 ± 0.263 1.397 ± 0.517	0.189 ± 0.107 0.150 ± 0.084 0.113 ± 0.111 0.137 ± 0.128 0.161 ± 0.120	0.90–2.31 1.17–2.78 1.11–2.40 0.46–1.89 0.30–2.78	0.162–0.217 0.129–0.171 0.085–0.142 0.104–0.170 0.148–0.175	< 0.001 < 0.001 < 0.001 < 0.001 < 0.001

*AS-OCT* anterior segment optical coherence tomography; *95% CI* 95% confidence interval; *pIOL* phakic intraocular lens

Subsequently, a generalized estimating equation (GEE) model was developed. In this model, we used the average of four repeated analyses (each analysis was acquired twice by both the first and the second observer) of the different distances with the average AS-OCT measurements as the dependent variable and the average Scheimpflug measurements as the independent variable. To assess the effect of the position of the measurement on this comparison, the same model was repeated with “position” as the fixed factor. Following this model, the measurements of the two devices were correlated with the standardized regression coefficient (*r*) of 0.962 (*p* < 0.001), with larger distances being measured by AS-OCT than by Scheimpflug imaging. Linear regression analysis yielded the following correlation (Eq. 1: correlation of AS-OCT and Scheimpflug for pIOL-to-endothelium distance measurements):

1 $$ {D}_{\mathrm{AS}-\mathrm{OCT}}=0.962\times {D}_{\mathrm{Scheimpflug}}+0.212\ \mathrm{mm} $$

where *D* is the pIOL-to-endothelium distance (in millimeters)

**Fig. 3:**
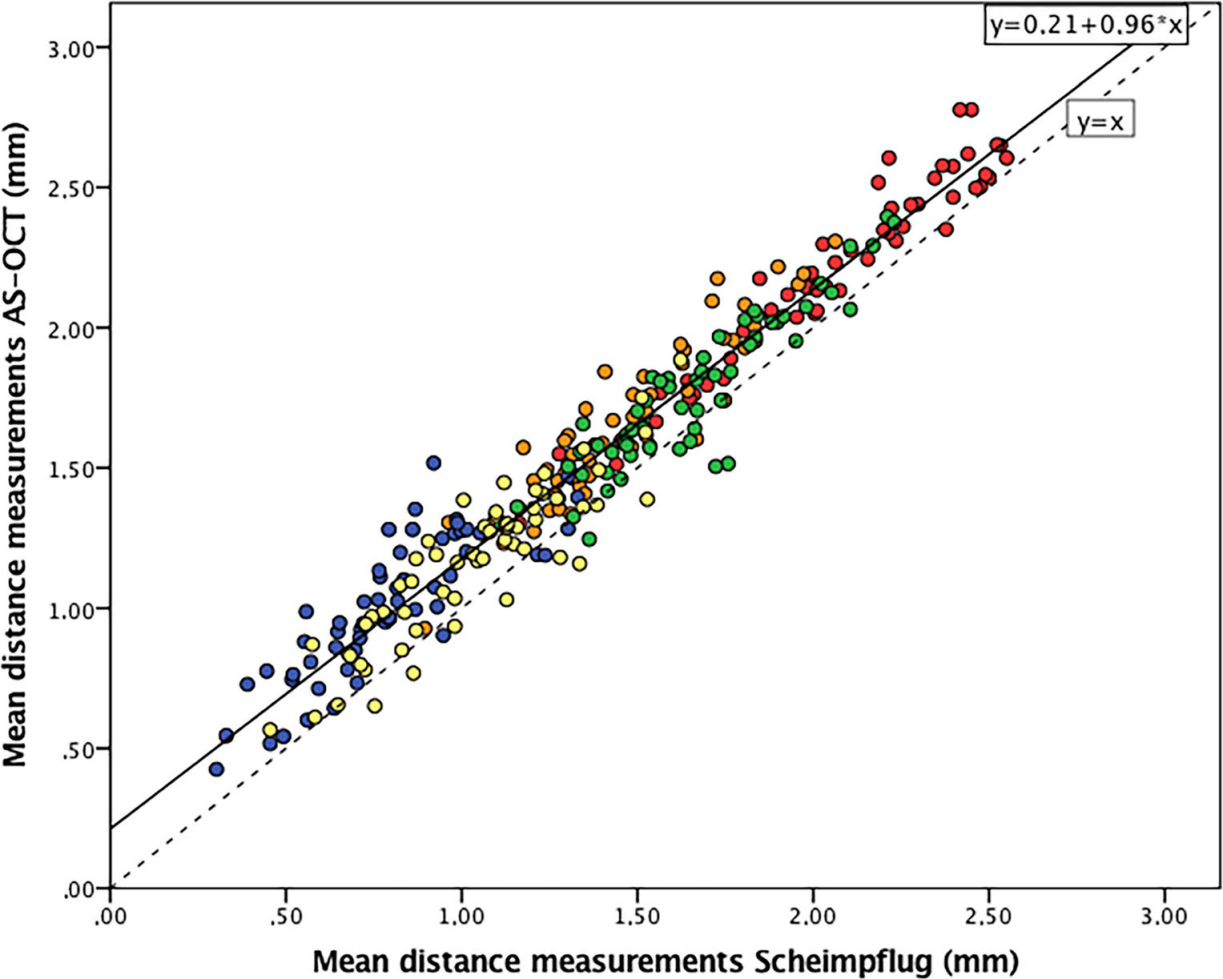
Scatter plot of the anterior segment optical coherence tomography (AS-OCT) measurements against Scheimpflug imaging measurements. The regression fit line (*black line*) following the relationship of the devices consistently shows higher measurements of AS-OCT compared with the *dashed line* which represents the absolute agreement of the instruments. Dot colors represent the positions of distances from the pIOL to the endothelium: *red*: central; *green:* 2.5 mm temporal from the center; *orange:* 2.5 mm nasal from the center; *yellow:* 4.0 mm nasal from the center; *blue:* 4.0 mm nasal from the center

This relation is clearly visible in the scatter plot of Fig. 3. To assess if this “overall” regression coefficient accounts for all distance positions separately, each regression coefficient of a position was compared with the average regression coefficient of the other positions using linear regression. For every clearance distance position, the regression coefficient did not significantly differ from the others, indicating that there was no effect of the different “distance position” slopes.

## Discussion

Correct positioning of an IF-pIOL in the anterior chamber is of high importance to determine long-term safety, as a smaller ACD and smaller distance from the edge of the pIOL to the endothelium can cause accelerated EC loss, which could lead to the need for early pIOL removal [19, 22]. Jonker et al. have recently reported a prevalence of IF-pIOL explantation due to excessive EC loss of up to 6.0% during 5- and 10-year follow-up [19]. Today, both AS-OCT and the Scheimpflug imaging are used to measure the pIOL edge to endothelium distance before and after pIOL implantation [11, 13, 15, 18]. The overall reproducibility of ACD biometry before and after pIOL implantation has been documented for both imaging modalities [23, 24], and a comparison study for ACD has shown a significant difference between the AS-OCT and Scheimpflug [23]. However, no reproducibility or comparison studies of the pIOL edge to endothelium distance measured with these two different imaging modalities have been performed. In this study, we demonstrate good inter- and intra-observer reproducibility for AS-OCT and Scheimpflug imaging when performing these measurements. A comparison between the two modalities, however, shows a significant difference in the measurement of the pIOL edge to endothelium distance, with the AS-OCT measurements being consistently larger than the Scheimpflug measurements.

Let us take a brief look at the aspects that differ between these instruments: firstly, the Pentacam HR, which uses Scheimpflug imaging, provides good images of the anterior segment. However, complex geometrical adjustments are performed to correct optical distortions caused by this modality [25, 26]. With AS-OCT, these optical corrections do not need to be made for axial measurements. However, for peripheral measurements, refraction at the corneal surface will result in a systematic error [27]. Moreover, based on this study, similar differences between OCT techniques, such as spectrometer-based and swept-source OCT, are plausible as these use different optical setups [28] which might result in similar systematic differences in apparent pIOL-to-endothelium distances. Secondly, we need to consider the effect of the different software instructions to measure the pIOL-to-endothelium distance: With the Pentacam software, minimum pIOL-to-endothelium distances are *automatically* identified and visualized for different positions after aligning the 3-D pIOL template. By contrast, the OCT calculations are based on *manually* defined distances since both the pIOL template and all the different distances are manually dragged and drawn (vector tool) onto the 2-D anterior segment scan. Although this manual interaction could reduce the inter- and intra-observer reproducibility, especially for less trained operators, it cannot explain the systematic difference between both devices.

Different models and minimum (“critical”) pIOL-to-endothelium distances are described in the literature for monitoring anterior chamber pIOL safety. Baikoff [11] at first suggested a minimum safety distance between the pIOL and corneal endothelium of 1.5 mm, a distance based on Scheimpflug results from earlier studies [11, 17]. Doors et al. [12, 13] evaluated pIOL clearances with the Visante OCT. Ferreira et al. [22] provided the clinicians with a new safety reference in 2014: a minimum *central* clearance distance of 1.7 mm, based on their Pentacam results. Recently, Jonker et al. [19] have demonstrated a 10.3% EC loss over 5 years and 20.5% over 10 years with a mean distance between the central pIOL edge and endothelium of 2.17 mm using the Visante AS-OCT. This risk showed a linear increase in EC loss with smaller distances.

For correct interpretation of the previously mentioned “critical minimum pIOL-to-endothelium distance,” including the risk of EC loss, the imaging modality used to obtain the pIOL-to-endothelium distance should be taken into account, as, according to our results, AS-OCT overestimates this distance compared with Scheimpflug. When using a Scheimpflug-based minimum safety distance for an AS-OCT scan, we suggest the use of our conversion equation. For example, based on Eq. 1, the minimum safety distance should be 1.84 mm, instead of 1.7 mm as proposed by Ferreira [22], when using AS-OCT. This difference of 0.14 mm is relevant for the follow-up of the patients, as it could explain increased EC loss. It is, however, important to realize that the found relation between both devices, and therefore also the modified safety distance, is not only vendor but also potentially software version–dependent.

In conclusion, AS-OCT and Scheimpflug imaging measuring the distance from the anterior edge of a pIOL to the corneal endothelium are both accurate with good reproducibility, but the AS-OCT provides consistently larger measurements compared with Scheimpflug imaging. This difference is of great clinical importance for the follow-up of pIOL positioning in the anterior chamber. We therefore suggest not to use these two imaging modalities interchangeably for measuring the pIOL-to-endothelium distance during follow-up. Clinicians using a fixed minimum safety distance or predictive model for safety follow-up should be aware of the instrument used for measurement as conversion might be needed.

## Electronic supplementary material

ESM 1 Bland-Altman plots showing the difference in distance measurements between the anterior segment optical coherence tomography and Scheimpflug imaging modalities for (a) central, (b) 2.0 mm nasal, (c) 2.0 mm temporal, (d) 4.0 mm nasal, and (e) 4.0 mm temporal of the anterior edge of the pIOL to the endothelium. The red line represents the mean, the black line the upper and lower 95% confidence interval, the dashed lines the upper and lower 95% limits of agreement (LoA). *Triangles*: hyperopic eyes; *dots*: myopic eyes. (PNG 112 kb)

High Resolution image (TIF 7251 kb)

## Data Availability

The data that support the findings of this study are available from the corresponding author, ZSG, upon reasonable request.
