# Supplementary figures and images for: Differences between Scheimpflug and optical coherence tomography in determining safety distances in eyes with an iris-fixating phakic intraocular lens
Source: Graefes Arch Clin Exp Ophthalmol. 2020 Aug 6;259(1):231–8. doi: 10.1007/s00417-020-04874-7 (PMC7790774; doi:10.1007/s00417-020-04874-7)

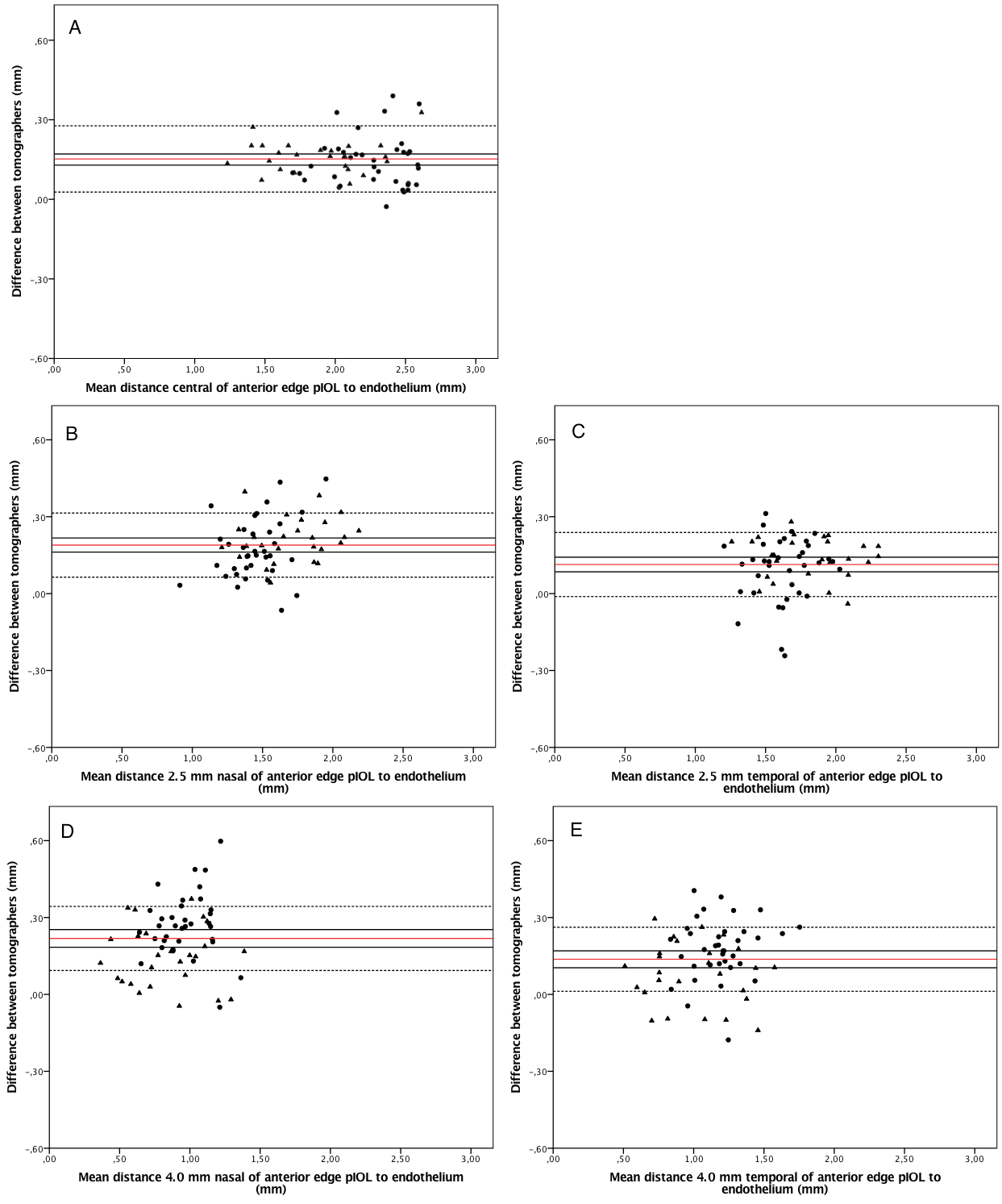

Supplement: Supplementary file 1 — Bland-Altman plots showing the difference in distance measurements between the anterior segment optical coherence tomography and Scheimpflug imaging modalities for (a) central, (b) 2.0 mm nasal, (c) 2.0 mm temporal, (d) 4.0 mm nasal, and (e) 4.0 mm temporal of the anterior edge of the pIOL to the endothelium. The red line represents the mean, the black line the upper and lower 95% confidence interval, the dashed lines the upper and lower 95% limits of agreement (LoA). Triangles: hyperopic eyes; dots: myopic eyes. (PNG 112 kb) [file 417_2020_4874_Fig4_ESM.png]

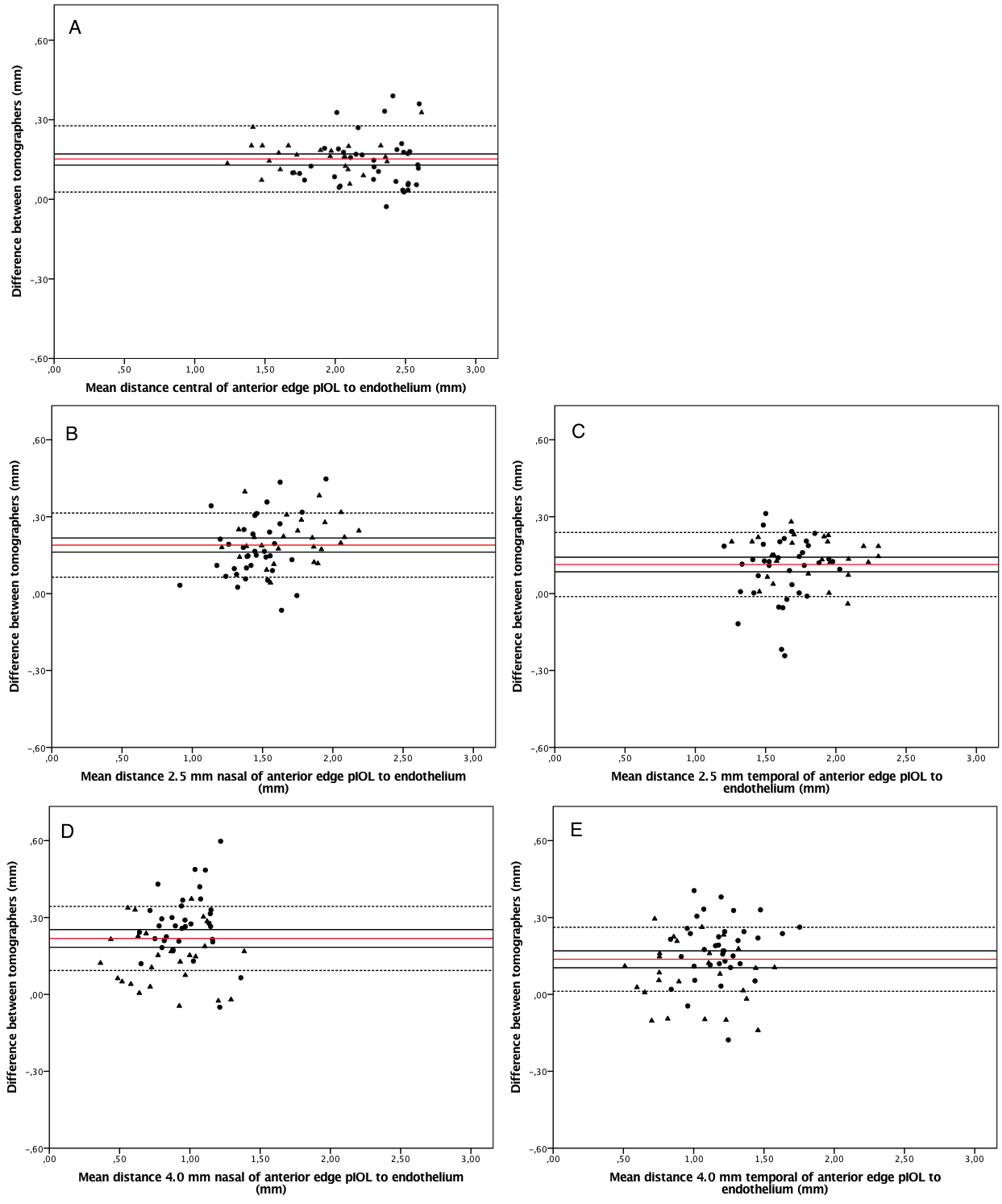

Supplement: Supplementary file 2 — High Resolution image (TIF 7251 kb) [file 417_2020_4874_MOESM1_ESM.tif]
